# Evaluation of a universal early intervention for parents and children from birth to age five

**DOI:** 10.1093/eurpub/ckac131.247

**Published:** 2022-10-25

**Authors:** F Sampaio, J Häggström, R Ssegonja, E Eurenius, A Ivarsson, AM Pulkki-Brännström, I Feldman

**Affiliations:** 1 Department of Public Health and Caring Sciences, Uppsala University, Uppsala, Sweden; 2 Department of Statistics, Umeå University, Umeå, Sweden; 3 Department of Medical Sciences, Uppsala University, Uppsala, Sweden; 4 Department of Epidemiology and Global Health, Umeå University, Umeå, Sweden

## Abstract

**Background:**

This study aimed to investigate the health and economic outcomes of a universal early intervention for parents and children, the Salut Programme, from birth to when the child completed five years of age.

**Methods:**

This study adopted a retrospective observational design using routinely collected register data with respect to both exposures and outcomes from a county in northern Sweden. Areas that received care-as-usual (non-Salut area) were compared to areas where the Programme was implemented after 2006 (Salut area) in terms of: i) health outcomes, healthcare resource use and related costs around pregnancy, delivery and birth, and ii) healthcare resource use and related costs, as well as costs related to care of sick child. Costs were estimated for inpatient care and specialised outpatient care for mothers and children. Two analyses were conducted: a matched difference-in difference analysis using the total sample and an analysis including a longitudinal subsample.

**Results:**

The longitudinal analysis on mothers who had given birth in both the pre- and post-measure periods showed that those that had been exposed to the Salut Programme, had on average 6% (95% CI 3-9%) more full-term pregnancies and 2% (95% CI 0.03-3%) more babies born within normal weight range, compared to mothers who had only care-as-usual. Savings were incurred in terms of outpatient care related costs for children of mothers in the Salut area ($826). The difference-in-difference analysis using the total sample did not result in any significant differences in health outcomes or cumulative resource use over time.

**Conclusions:**

The Salut Programme achieved health gains at a reasonable cost for children and parents, and may lead to lower usage of outpatient care. Other indicators point towards positive effects but the small sample size may have led to underestimation of true differences. The current findings support the continuous investment in this early childhood programme.

**Key messages:**

• The Salut Programme improves the health of children and parents at a low cost.

• The Salut Programme as a health promotion early intervention is value for money and should be included in the local policy investment agenda.

